# Unusual Presentation of Pseudoaneurysm with Trochanteric Fracture Femur with Associated Long-Term Antiepileptic Therapy

**DOI:** 10.1155/2014/896968

**Published:** 2014-08-12

**Authors:** Nipun Rana, Gajanand Dhaked, Satish Sharma, Sandeep Tripathi

**Affiliations:** Department of Orthopedics, Sir Ganga Ram Hospital, New Delhi 110060, India

## Abstract

Arterial injury following impalement due to a trochanteric hip fracture-fragment per se has been documented rarely. We report a case of pseudoaneurysm of profunda femoris artery at the first perforator branch in a 48-year-old male, with trochanteric hip fracture following a fall during an epileptic episode. Persistent recalcitrant pain, globular swelling in the groin, unexplained drop in the haemoglobin level, and color doppler ultrasonography findings were salient features to the diagnosis. Additionally, we collected all reported cases of pseudoaneurysm associated with hip fractures. We reviewed the literature regarding the incidence, treatment, and prognosis for the same. Acute vascular injury was probably caused by the spikes of fractured lesser trochanter which was found to be displaced superomedially. All trochanteric fractures especially those with displaced lesser trochanter fragment should be closely watched for the possibility of vascular injury. Early diagnosis and treatment in a staged manner can prevent the catastrophic vascular event and hence the limb.

## 1. Introduction

Arterial injuries in the vicinity of proximal femur have been reported after total hip replacement, DHS fixation [1, 2], nail-plate fixation [3, 4], external fixation, and gamma nailing [5, 6] for proximal femoral fractures. An arterial injury following impalement due to a trochanteric hip fracture-fragment per se has been rarely documented [5, 7–10]. We share our experience in one case with intertrochanteric fracture, two week following his development of an intramuscular hematoma and pseudoaneurysm emphasizing the need for a high-grade clinical suspicion for such vascular injuries while dealing with hip fractures.

## 2. Case Presentation

**Figure 1:**
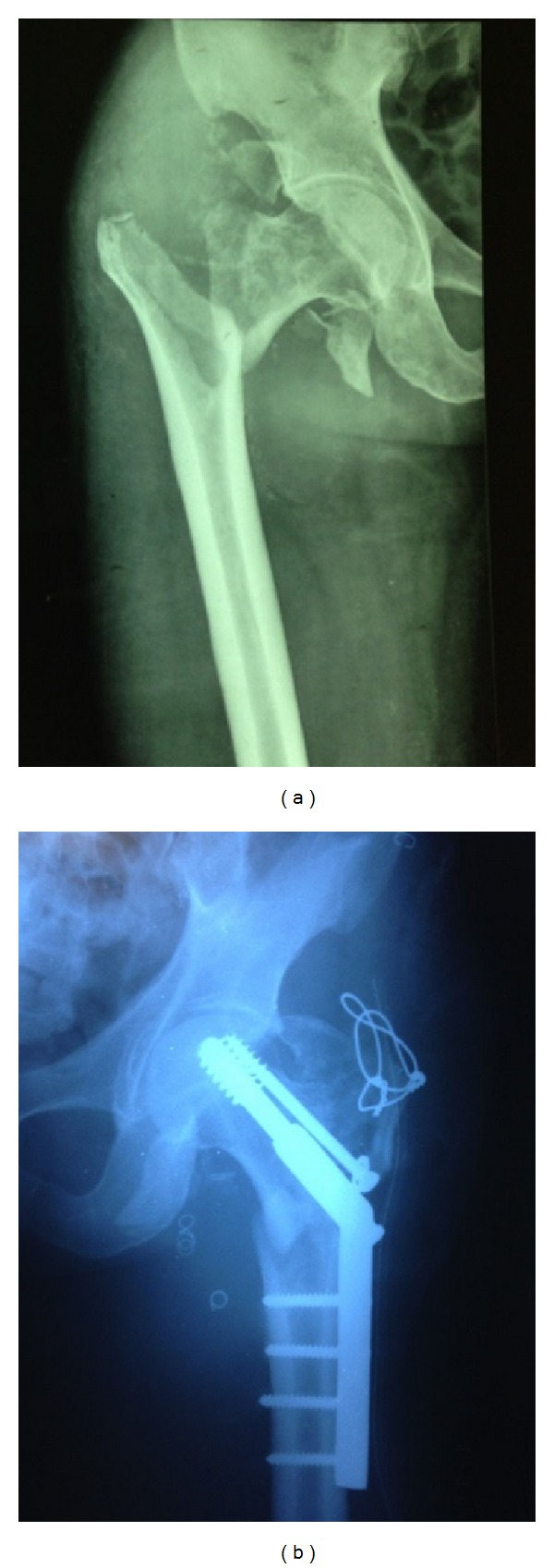
Conventional radiographs of fracture, coiling, and fixation. (a) Showing the inter trochanteric fracture with greater trochanter fracture right hip and (b) showing the endovascular repair done with coiling first and later dynamic hip screw (DHS) fixation and tension band wiring.

48-year-old, tall, and slim male had a fall at home following an attack of seizures, with resultant injury to the right hip region leading to pain and inability to bear weight on the same side. Prior to injury the man could walk normally. He did not suffer from any other comorbidities. His pedal pulses were palpable and showed no signs of ischemic injury to the limb. The plain radiograph of the right hip showed a displaced unstable intertrochanteric fracture right hip (Figure 1(a)). Surgical fixation of the fracture was deferred considering his neurological condition; hence, he was managed conservatively with boot and bar application with traction onto right foot. He declined the use of plaster boot due to claustrophobia and cut open the cast himself. He was then put on skeletal traction through an upper tibial skeletal pin.

On day 14 following the injury, he complained of severe excruciating pain in the anteromedial aspect of the right thigh, below the inguinal region with an associated globular swelling over the same location, of the size of a cricket ball, not associated with any appreciable thrill and bruit. He gives history of swelling showing diurnal variation, with an increased size in the morning. He was managed conservatively with pain killers and hot fomentation, considering it to be an organized hematoma. In the following 2 months, there was an improvement in the pain over size of swelling, with a more worrisome developing pallor. The hemoglobin levels were found to drop progressively, pondering to find the cause for it.

Two and a half months following the injury he was referred to a tertiary care centre. Color Doppler ultrasonography right thigh revealed a pseudoaneurysm arising from profunda femoris artery and its branches. Fresh radiograph revealed a right sided, nonunited intertrochanteric fracture with a greater tuberosity fracture missed initially.

**Figure 2:**
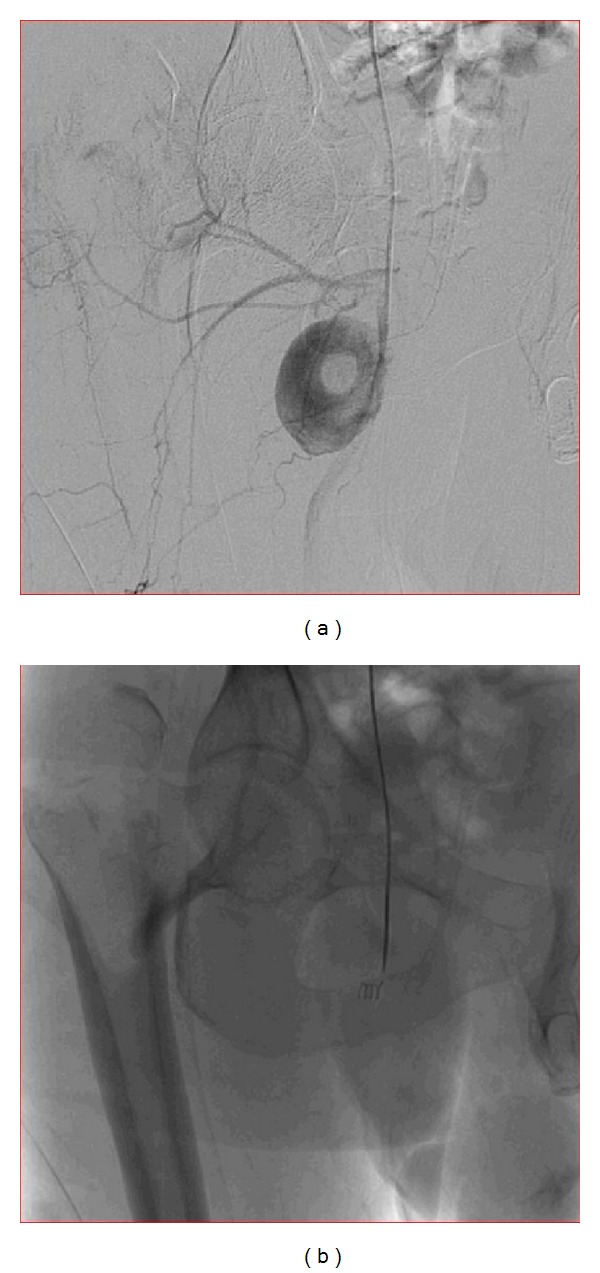
(a) Shows the conventional angiography and the filling up of the dye femoral vessels and the associated aneurysm. (b) Shows the insertion and application of the endovascular coil at the mouth of the aneurysm with successful repair of the aneurysm and the dye not entering the aneurysmal void.

He was managed in a staged fashion. First, to begin with, an endovascular repair of aneurysm arising from profunda femoris artery was done (Figure 1(b)). Conventional angiography (Figure 2) revealed a pseudoaneurysm located over the posteromedial aspect of the femur though a posterolateral location has also been reported in a case of proximal femoral nailing for intertrochanteric femoral neck fracture [11].* There were no symptoms of peripheral ischemia following the surgery*. A week later he was treated for the hip fracture with a DHS fixation with TBW (tension band wiring) with added autologous bone grafting (Figure 1(b)). Postoperatively on day 2 the drain was removed and patient was mobilized out of the bed. Nonweight bearing walker aided ambulation was begun. He was refrained from weight bearing on operated limb and crossed leg maneuvers. The patient is under followup at present, pain free, and walking unaided.

We bother the readers to note the possible occurrence of such aneurysms in proximal femur fracture, which need not mandatorily be of early onset. An association of seizure disorder and its medications (tab. valproate 500 mg twice daily and tab. eptoin 100 mg thrice daily dosage) with such an osseovascular traumatic event is unlikely but does need further investigation.

## 3. Discussion

The case under study showed a subacute presentation of 2 weeks following injury with the probable cause as vascular injury due to spike of fractured lesser trochanter. Delayed presentation is usually secondary to prolonged impingement or erosion of the artery by a protruding fixation screw particularly seen in arteries with atherosclerotic plaques [12].

The arteria profunda femoris is a large branch of femoral artery arising 3.5 cm distal to the inguinal ligament (Figure 2). It spirals posterior to the femoral vessels to medial side of femur passing distally between adductor muscles, finally piercing through the adductor magnus posteriorly to anastomose with the upper muscular branches of the popliteal. This terminal branch is also sometimes named as fourth perforating artery. There are usually* three perforating branches* arising from profunda femoris which are in close relation to the femur as they traverse distally. They pass under the linea aspera under small tendinous arches and issue muscular, cutaneous, and anastomotic branches. The* first perforating artery* passes back between the pectineus and adductor brevis (sometimes through the latter), piercing the adductor magnus near the linea aspera, anastomosing with the inferior gluteal, medial, and lateral circumflex and second perforating artery (cruciate anastomosis). The first perforating artery is the usual site for injury.

Bony injury is more likely to occur to common femoral artery and the superficial femoral artery as profunda femoris lies deep in the thigh and is protected by the vastus medialis muscle cover [13]. The above statement is disapproved by another author [14] who believed profunda femoris was more often involved than the superficial femoral vessels and the most common mechanisms of injury were pressure of sharp bone fragment (lesser trochanter), tip of protruding cortical screw (extramedullary implant), and less often by the drill bit or incorrectly placed elevators. In one study they saw coagulopathy as a result of platelet dysfunction in patients with myelodysplasia as a cause to pseudoaneurysm formation [13]. In our patient the coagulation profile was normal except for raised d-dimer levels which could be explained by recent surgery. Any correlation between the seizure disorder and its medications with the vascular insult shall require further investigation and research which is out of scope of this paper.

A pseudoaneurysm may be present as a local swelling, simulating a local soft tissue hematoma, inguinal lymphadenopathy, infected granuloma, DVT, or a soft tissue sarcoma. Pulsatility and bruit detection may be difficult because of the deep location [12]. In our patient initial evaluation did not revealed pulsatility or bruit and hence was underdiagnosed as a local hematoma. This led to delay in diagnosis. The factors for early suspicion are an increasing size of swelling not subsiding with local conservative measures like anti-inflammatory drugs or hot fomentation, an excruciating pain not explained by traumatic event and progressive decline in hemoglobin levels with normal or high normal cell count. Changes in color and temperature of the limb and the presence and strength of the pedal pulses have not been proven to be helpful in diagnosis unless a large artery is involved [12].

In high suspicion cases one should investigate further with invasive or noninvasive radiological techniques. We found that in emergency department noninvasive technique like Duplex ultrasonography was adequate enough to diagnose a pseudo aneurysm. In color Doppler ultrasound scans, pseudoaneurysm has a to and fro pattern due to high rate of inflow and outflow of the blood, which is also known as the “yin-yan” sign [12].

The standard treatment today is femoral angiography and percutaneous endovascular (transarterial) embolization with coils of the truncated muscular branches and the perforators of the profunda femoris artery. An emergency procedure in a hemodynamically unstable patient would be to do an emergency exploration and clot evacuation for large hematomas as they can cause compartment syndrome of thigh [12]. In our patient endovascular repair was done as the patient was hemodynamically stable and had involvement of a branch of profunda femoris artery only. Removal of the bone fragment showing impingement has been noted in one study and importance of this step has not been found essential in our case [8].

## 4. Conclusion

Femoral vessel injury following an intertrochanteric fracture before any surgical intervention is a possibility though rare, which may present weeks to months from the time of injury and hence may lead to delay in diagnosis. Treatment should be directed in a staged manner to save the circulation first and then manage the fracture. Percutaneous (transarterial) coil embolization is the treatment of choice for smaller aneurysms and otherwise haemodynamically stable cases. We chose embolization in the first stage so as to prevent any inadvertent event from occuring while performing fixation in the second stage.

## References

[1] Kleintz R, Nolte U (1993). Development of aneurysma spurium of the arteria profunda femoris as a late complication of DHS osteosynthesis. *Unfallchirurg*.

[2] Murphy PG, Geoghegan JG, Austin O, More-O'Ferrall R, Quinlan WR, Keaveny TV (1999). Pseudoaneurysm of the profunda femoris artery due to intertrochanteric fracture of the hip. *Archives of Orthopaedic and Trauma Surgery*.

[3] Ebong WW (1978). False aneurysm of the profunda femoris artery following internal fixation of an intertrochanteric femoral fracture. *Injury*.

[4] Wang C (1975). False aneurysm of the profundus femoral artery following nail-plate fixation for intertrochanteric fracture of the hip. *The Journal of the Medical Society of New Jersey*.

[5] Lohmann H, Esenwein S, Geier B, Vogel T, Kleinert H (2009). False aneurysm of the deep femoral artery due to pertrochanteric fracture of the hip with displaced fragment of the lesser trochanter. *Zeitschrift für Orthopadie und Unfallchirurgie*.

[6] Yang KH, Park HW, Park SJ (2002). Pseudoaneurysm of the superficial femoral artery after closed hip nailing with a Gamma nail: report of a case. *Journal of Orthopaedic Trauma*.

[7] Obry C, Merti P, Woestelandt T, Vives P (1988). False aneurysm of the profunda femoris artery following an intertrochanteric fracture of the femur: a case report. *Revue de Chirurgie Orthopedique et Reparatrice de l'Appareil Moteur*.

[8] Ritchie ED, Haverkamp D, Schiphorst TJMJ, Bosscha K (2007). False aneurysm of the profunda femoris artery, a rare complication of a proximal femoral fracture. *Acta Orthopaedica Belgica*.

[9] Fernandez Gonzalez J, Terriza MD, Cabada T, Garcia-Araujo C (1995). False aneurysm of the femoral artery as a late complication of an intertrochanteric fracture: a case report. *International Orthopaedics*.

[10] O'Donoghue D, Muddu BN, Woodyer AB (1994). False aneurysm of the profunda femoris artery due to malunion of a hip fracture. *Injury*.

[11] Rajaesparan K, Amin A, Arora S, Walton NP (2008). Pseudoaneurysm of a branch of the profunda femoris artery following distal locking of an intramedullary hip nail: an unusual anatomical location. *HIP International*.

[12] Chan WS, Kong S, Sun K, Tsang P, Chow H (2010). Pseudoaneurysm and intramuscular haematoma after dynamic hip screw fixation for intertrochanteric femoral fracture: a case report. *Journal of Orthopaedic Surgery*.

[13] Cowley A, Williams D, Butler M, Edwards A, Parsons S (2007). Pseudo-aneurysm of the profunda femoris artery as a late complication of hip fracture in a patient with myelodysplasia.. *Annals of the Royal College of Surgeons of England*.

[14] Bartonicek J (2009). Injuries to the femoral vessels in fractures of the hip. *Rozhledy v Chirurgii*.

